# Systematic Literature Review Regarding Heart Rate and Respiratory Rate Measurement by Means of Radar Technology

**DOI:** 10.3390/s24031003

**Published:** 2024-02-04

**Authors:** Magdalena Liebetruth, Kai Kehe, Dirk Steinritz, Stefan Sammito

**Affiliations:** 1 German Air Force Centre of Aerospace Medicine, 51147 Cologne, Germany; 2 Department of Occupational Medicine, Faculty of Medicine, Otto von Guericke University of Magdeburg, 39120 Magdeburg, Germany; 3 Bundeswehr Medical Service Headquarter, Department A-VI Public Health, 56072 Koblenz, Germany; 4 Bundeswehr Institute of Pharmacology and Toxicology, 80937 Munich, Germany

**Keywords:** radar technology, breathing, heart rate, vital parameters

## Abstract

The use of radar technology for non-contact measurement of vital parameters is increasingly being examined in scientific studies. Based on a systematic literature search in the PubMed, German National Library, Austrian Library Network (Union Catalog), Swiss National Library and Common Library Network databases, the accuracy of heart rate and/or respiratory rate measurements by means of radar technology was analyzed. In 37% of the included studies on the measurement of the respiratory rate and in 48% of those on the measurement of the heart rate, the maximum deviation was 5%. For a tolerated deviation of 10%, the corresponding percentages were 85% and 87%, respectively. However, the quantitative comparability of the results available in the current literature is very limited due to a variety of variables. The elimination of the problem of confounding variables and the continuation of the tendency to focus on the algorithm applied will continue to constitute a central topic of radar-based vital parameter measurement. Promising fields of application of research can be found in particular in areas that require non-contact measurements. This includes infection events, emergency medicine, disaster situations and major catastrophic incidents.

## 1. Introduction

The measurement of vital parameters allows for accurate, non-invasive registration and monitoring of a person’s state of health. The four main vital parameters include the heart rate, respiratory rate, body (core) temperature and blood pressure [1]. Since they are strongly dependent on the general health status of a person and react to fever, stress and infections [2,3], among other things, they are suitable for identifying acute health problems or for monitoring patients over an extended period of time. The detection of heart and respiratory rates is usually effected via contact electrodes used for taking an electrocardiogram (ECG) and via respiratory belts or indirectly by photoplethysmography within the scope of the measurement of peripheral oxygen saturation. These methods, however, may cause irritation and, possibly, pain and infections [4]. The corresponding values are indicated in beats per minute for the heart rate and in breaths per minute for the respiratory rate. Due to the context, both values are abbreviated as bpm (breaths/beats per minute).

Radar technology can be used for non-contact measurement of these central, vital parameters. As can be seen from the current example of the COVID-19 pandemic, the possibility of non-contact measurement has many advantages, especially in the field of pandemic control and in the prevention of the spread of infectious diseases [5]. The first measurements using radar technology were performed in the 1970s [6,7]. Since then, the prospect of countless advantages of non-contact measurement has induced scientific advancements in the field of radar-based vital parameter acquisition [8], which mainly take place in the technical area. In order to do justice to this great interest and the large number of publications, this systematic literature review analyzes the current literature to clarify the state of the art of heart and respiratory rate measurement using radar technology from a medical point of view and shows what is required for or what prevents a broader application.

## 2. Materials and Methods

This systematic literature review was conducted in accordance with the standard of the PRISMA (Preferred Reporting Items for Systematic Reviews and Meta-Analyses) [9] statement. For this purpose, the search query ((“Heart rate” OR “heart” AND “rate” OR “HR”) OR (“EKG”) OR (“breath”) or (“vital parameter”) AND (“radar” OR “microwave”)) was applied in the PubMed, German National Library, Austrian Library Network (Union Catalog), Swiss National Library and Common Library Network databases on 29 November 2022. The inclusion criterion was a reference to non-contact measurement of heart and respiratory rates using radar technology. Exclusion criteria were publication in a language other than English, measurements by means of contact radar (so-called “wearables”), radar as an influencing parameter instead of a measuring method and exclusive measurement of other vital parameters such as heart rate variability (HRV) or “microwave resonators” as radar keys. In accordance with scientific practice, withdrawn articles were not included.

**Figure 1:**
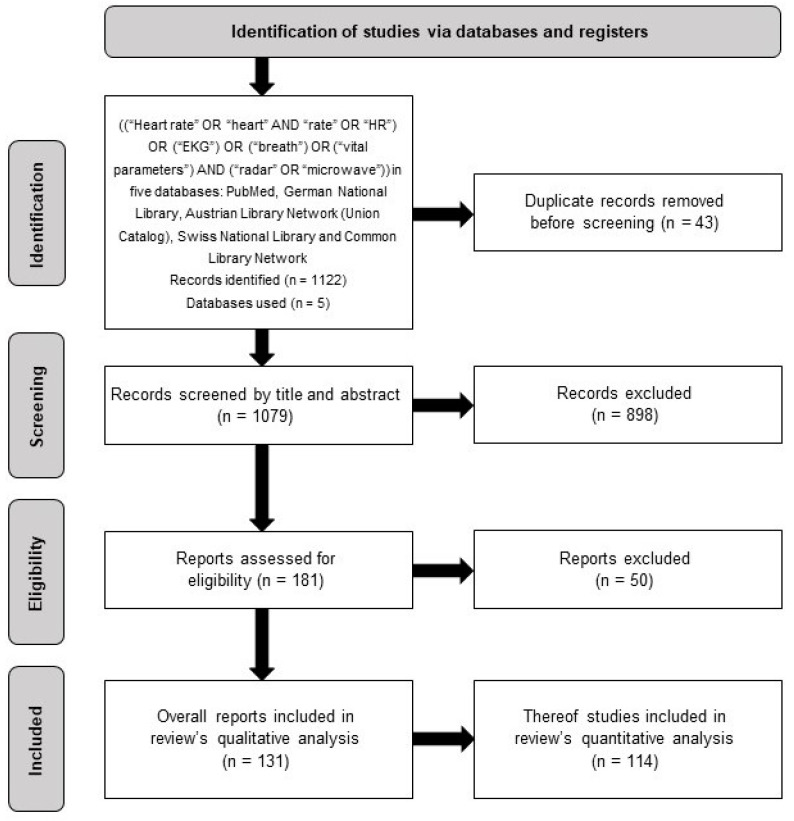
Flow of information through the different phases of the systematic review.

The search query yielded a total number of 1122 hits. After 43 duplicates had been removed, 1079 of these hits were included in the preselection. After screening the titles and abstracts, 898 articles were excluded so that 181 full texts were assessed for eligibility. Of these, an additional 50 articles had to be excluded. Thus, a total of 131 sources were included in the qualitative evaluation, 114 of which contained an experimental part and were also taken into account quantitatively. The term “experimental part” includes all articles containing a test part, irrespective of the weighting of the “conduct of a practical test” and “theoretical execution” parts. Figure 1 shows the associated PRISMA flowchart of the review process. In the following presentation of the results, only heart and respiratory rates were taken into account, since the focus of this paper is on these two vital parameters. If other vital parameters were acquired as well, they are not indicated.

## 3. Results

**Figure 2:**
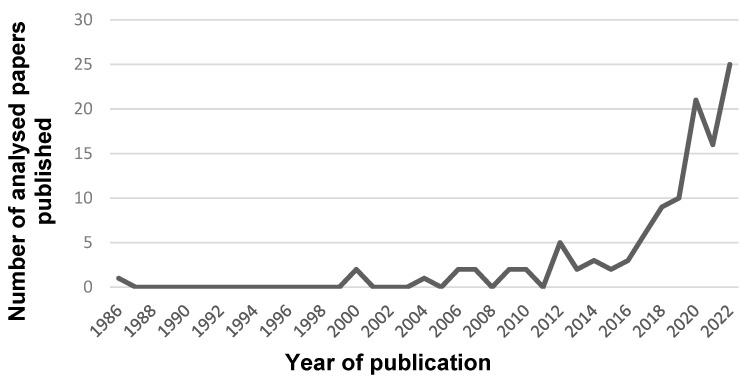
Number of released sources analyzed in this review per publication year.

A total of 114 of the evaluated articles contained experimental parts. In these experimental parts, different radar types were used for measuring the vital parameters. Looking at the years of publication of the studies, it was noticeable that there was a clear upward trend in the number of publications per year. This can be seen in Figure 2. In total, 88 of these publications were original studies, while 26 publications were obtained from conference proceedings.

Table 1 lists the titles depending on whether the radar used belonged to the group of continuous wave (CW) radars (*n* = 58), frequency-modulated continuous wave (FMCW) radars (*n* = 24) or ultra-wideband (UWB) radars (*n* = 34). In three papers, different radar types were used [10,11,12]. For one study, it was not possible to make an assignment [13]. In Table 1, the titles are grouped in accordance with the radar types used, and within each category (group), they are sorted by the vital parameters measured.

In 74 studies, both the heart rate and the respiratory rate were measured [2,5,10,12,14,15,16,17,18,19,20,21,22,23,24,25,26,27,28,29,30,31,32,33,34,35,36,37,38,39,40,41,42,43,44,45,46,47,48,49,50,51,52,53,54,55,56,57,58,59,60,61,62,63,64,65,66,67,68,69,70,71,72,73,74,75,76,77,78,79,80,81,82,83]. In 14 studies, only the respiratory rate was considered [3,11,13,84,85,86,87,88,89,90,91,92,93,94], and in 26 studies, only the heart rate [95,96,97,98,99,100,101,102,103,104,105,106,107,108,109,110,111,112,113,114,115,116,117,118,119,120]. Table 2 shows the number of acquisitions of the vital parameters considered in the experiments in relation to the radar type. Deviations from the numbers indicated in the text result from the use of several radar types per study.

**Table 1:** Summary of the systematic literature review.

Study	Measured Parameters		Subjects ^1^	Number and Position of Subjects per Measurement	Position: Radar towards Person	Distance Radar–Person [m]	Radar [GHz]	Ref.	Focus	Quantitative Results
**Continuous Wave (CW) Radar Studies**										
[2]	RR	HR	8	1: laid	sideways, below	0.2	10 (RR) 24 (HR)	C	a	ambulance standing r(HR) = 0.76 r(RR) = 0.98 ambulance driving r(HR) = 0.69 r(RR) = 0.97
[3]	RR		3	1: seated, standing, laid	frontal above	2–3	24.125	C	a	deviation (RR) = 1.88 bpm
[14]	RR	HR	n/a	1: seated, working out on an ergometer	sideways	0.4	24	C	a	accuracy (HR) = 99% (RR) = 98%
[15]	RR	HR	n/a	1: seated	frontal	1.5	10	C + breathing after rhythm of a metronome	a	deviation depending on conditions (HR) = 0.87–1.25%, (RR) = 2.14–4.86%
[16]	RR	HR	5	5: seated	frontal	1	2.4	C	a	deviation (HR) = 1.71 bpm deviation (RR) = 2.28 bpm
[17]	RR	HR	9 + 3 hospitalised infants	1: laid	dorsal	0.05	24	C	a	r(RR) = 0.83 and r(HR) = 0.96 RMSE (RR) = 1.66 bpm and RMSE (HR) = 1.94 bpm
[18]	RR	HR	n/a	n/a	frontal	0.5	24	subject holding his breath, C for HR	a	n/a
[19]	RR	HR	n/a	1: seated	frontal	0.5	2 × 5.8	C	a	97.8% accuracy (96.8% for respiration and 98.2% for heartbeat at 30°. 96% for respiration and 99.2% for heartbeat at 45°, and 98.4% for respiration and 96% for heartbeat at 60°)
[20]	RR	HR	2	1: seated	frontal	2	5.8	C	a	deviation (HR) = 1.13%, RR n/a
[21]	RR	HR	101 (47 dengue fever patients, 54 healthy students)	1: standing	frontal	n/a	24	C	a	98.1% precision in detecting illness
[22]	RR	HR	2	1: seated	frontal, dorsal	0.6–1	2.5	C	a	good measurement results without statistical analysis
[23]	RR	HR	7	1: laid	dorsal	0.1	24	C	a	correlation coefficient r(HR) = 0.92 und r(RR) = 0.99
[24]	RR	HR	1	1: seated	frontal	1.5	2.4	C	a	n/a
[25]	RR	HR	4	1 (+interference persons): seated	frontal	0.3	0.040	C	a	absolute deviation (HR) < 1 bpm, (RR) n/a
[26]	RR	HR	3	1: standing	frontal	0.3–0.5	10.6	C	a	(HR) < 10% in all conditions, (RR) n/a
[27]	RR	HR	7	1: standing	frontal	0.01/0.3	10	C		r (HR) = 0.97; r (RR) = 0.93
[28]	RR	HR	3	1: laid	dorsal, sideways	through bed	4 × 24	C		RMSE (HR) = 24 ms; (RR) = 200 ms
[29]	RR	HR	8	1: laid	above	<0.50, through glas	1215	C		r(HR) = 0.98, r(RR) = 0.86
[30]	RR	HR	1	1: seated	frontal	1/3/6/9/12/15/18/21/24	2.45	C		Qualitative: HR up to 18/RR up to 69 m measurable
[31]	RR	HR	n/a	1: seated behind wall, laid	frontal, dorsal, sideways	1–30 (through wall of 0.15 m: subject 0.6–5 m and radar 0.3 m from wall; without wall: subject in 30 m distance)	10	n/a		n/a
[32]	RR	HR	n/a	1 (+ interference person): laid	frontal	3 (through earthquake rubble)	1.15–0.45	holding breath		n/a
[33]	RR	HR	9	1: laid	below	0.15	24 + 10	C		n/a
[34]	RR	HR	n/a	1: laid	frontal	1	2.4	C		r(HR) = 0.98, r(RR) = 0.994
[35]	RR	HR	n/a	1: n/a	frontal	0.4	2.4	C		n/a
[36]	RR	HR	2	1: seated	frontal	0.6	24	counting breaths, C for HR		deviation (RR) = 4.47–19.59%, (HR) = 1.60–5.79%
[37]	RR	HR	? + model	1	frontal	0.3/1/1.5	94	n/a		n/a
[38]	RR	HR	16 (rabbits)	1	frontal	0.4	1215	C		r(HR) = 0.91; r(RR) = 0.96
[39]	RR	HR	92 (57 influenza patients)	1: standing	frontal (breathing), diagonally below (heart beat)	0.3	10 (RR); n/a (HR)	proving of method in previous work		Positive predictive value for infection: screening 93%, sensitivity 88%, negative predictive value: 82%, specificity: 89%
[40]	RR	HR	2	1, 2: seated	frontal	0.5	24	C		deviation in two-subject experiment (RR) < 8.6%; HR: n/a
[41]	RR	HR	70 (47 dengue fever patients)	n/a	n/a	n/a	10	C for HR, n/a for breathing		Correlation coefficient r(HR) = 0.98, (RR) n/a
[42]	RR	HR	1 + model	1: seated	frontal, dorsal	0.42	24	C		n/a
[43]	RR	HR	1	1: n/a	n/a	0.4	5.8	C		accuracy (HR) = 96.6% and (RR) = 97.3%
[44]	RR	HR	2 + model	1: laid	frontal	0.2–1.2	24	C		deviation (RR) = 1 bpm, (HR) = 3 bpm
[82]	RR	HR	30	1: laid	above	0.4	24	C	a	RMSE (RR) = 0.828 bpm, RMSE of RR interval (HR) = 26.07 ms
[83]	RR	HR	10	1: seated	frontal	0.2–0.4	2.4	C	a	deviation (HR) = 0.8 bpm and (RR) = 3.5 bpm
[84]	RR		12 (premature infants)	1: laid	above (frontal, dorsal, sideways)	0.5 (through plexi glas)	24	C	a	(RR) < 10% deviation for 87.2–97% of measurements depending on setup (RMSE (RR) = 6.38%)
[85]	RR		2 phantoms + 1 person	1–2 phantoms/phantom + person: phantom/seated	frontal	1.5	2.4	breathing after metronome, phantom	a	n/a
[86]	RR		1	1: seated	frontal, dorsal (0°, 30°, 60°, 90°)	0.2 - 0.7	2.4	C		(RR)= 87.34–89.6% accuracy
[94]	RR		52 (with stable chronic heart failure)	1: laid	sideways	1–2.5	5.8	C		r(RR) = 0.98 (deviation (RR) = 0.31 bpm)
[95]		HR	5	n/a	frontal, dorsal	0.8	2.4	C	a	71% of measurements < 10% (HR) deviation (4–6 bpm deviation)
[96]		HR	5	1: seated	frontal	0.3–0.8	24	C	a	deviation (HR) = 4.2% (seated without moving), 4.8% (seated and typing)
[97]		HR	10 + model	1: seated	frontal	0.5	5.8	C	a	deviation (HR) = ±2%
[98]		HR	212	1: seated	frontal	n/a	24	visual control of breathing movement, C for HR	a	n/a
[99]		HR	212	1: seated, laid	frontal, above	n/a	25	C	a	deviation lying (HR) = 1.93 ± 1.76 bpm, seated (HR) = 9.72 ± 7.86 bpm
[100]		HR	21	1: seated	frontal	0.75	24	C	a	deviation (HR) = 15%
[101]		HR	3	1: seated	frontal	0.8	24	C	a	on average 75% better results
[102]		HR	5	1: seated	frontal	1	24	C	a	deviation depending on conditions (HR) = 3.0–6.3%
[103]		HR	5	1: seated	frontal	1	2.6	C, metronome breathing	a	deviation (HR) = 2.6 bpm
[104]		HR	n/a	1: laid	above	through plexiglass shield	0.5–4 and 4–18	C		n/a
[105]		HR	6	1: laid	below	through bed	24	C		accuracy (HR) = 80–98% (average 88.5%)
[106]		HR	8	1: laid	below	through bed	2 × 24	C		r(HR) = 0.703
[107]		HR	6	1: laid	sideways	0.4	2.45	C		accuracy (HR) = 96.78%
[108]		HR	1	1: n/a	frontal	1	2.4/5.8/10/16/60	n/a		accuracy (HR) = 100%
[109]		HR	3	1: seated, walking	frontal	0.15–7	10.6	n/a		n/a
[110]		HR	4	1: seated, standing, walking, laid	frontal	0.1–0.6	285–315	C, camera		deviation (HR) = 1.51 bpm on the forhead
[120]		HR	3	1: laid	above	0.8	24	C	a	<10% for 70% of measurements
**Ultra-wideband (UWB) and CW radar studies**										
[11]	RR		1	1: seated	frontal	1	3	C		n/a
[12]	RR	HR	12	n/a	frontal, dorsal, towards carotid arteries/lying on skin	0.2–1	(1) bw 0.10–6.5 (2) 24.17	C	a	r(HR) = 0.89; r(RR) = n/a
**UWB radar studies**										
[5]	RR	pulse rate	33 (14 patients with persisting atrial fibrillation)	1: laid	sideways, 45° from above	0.5	6.5–8	palpitation through physician		intraclass correlation coefficient (RR) = 0.852
[45]	RR	HR	34 (babies)	1: laid	orthogonal frontal	0.35	XK200 (Xandar Kardian, New York, Canada)	C		concordance correlation coefficient (RR) = 0.95, CCC (HR) = 0.97
[46]	RR	HR	n/a	1: seated	frontal	1.5	2.4 (bw 2)	C	a	(RR) within 7%/(HR) within 3% deviation
[47]	RR	HR	3	1: seated	6 different angles	1.7	7.3 (bw 2.5)	C		(HR) = 89–95.7% accuracy, (RR) = 74.2–88.3%
[48]	RR	HR	8	1/2/3: seated	frontal	1, 2, 3, 4, 5	7.29 (bw 1.4)	C	a	deviation (RR) = 5.14% and (HR) = 4.87%; in multi-person experment deviation (HR) < 1.13 bpm and (RR) < 0.56 bpm
[49]	RR	HR	n/a	n/a	frontal	1	4.7 (bw 3.2)	C	a	Accuracy (HR) = 95%, (RR) = 100% for 93% of measurements
[50]	RR	HR	3	3: laid	above	2.3	10.35	C	a	deviation HR ± 2 bpm, RR ± 0.3 bpm
[51]	RR	HR	21 (15 obstructive sleep apnea patients)	1: laid	above	0.5	6.5–8	C		r(HR) = 0.927, r(RR) = 0.959
[52]	RR	HR	5	2: seated	frontal, dorsal	1–2	6.8	algorithm	a	comparison with algorithm
[53]	RR	HR	50	1: seated, laid	frontal	1.5	6.5–8	C		CCC (RR) = 0.925, CCC (HR) = 0.749
[54]	RR	HR	3	1: seated	dorsal	through seat	3.8 (bw 2)	C		deviation (HR) depending on condition = 1.82–39.4%; (RR) n/a
[55]	RR	HR	5	1 (+interference person): laid	frontal	1	7.29 (bw 1.4)	C	a	deviation (HR) = 1.32 bpm, (RR) = 0.65%
[56]	RR	HR	3	2/3: seated	frontal	1.85 (0.2 m bevor and 1.5 m after wall of 0.15 m)	NVA 6100 chip CMOS (bw 0.85–9.55)	algorithm	a	“results were 100% successful”
[57]	RR	HR	8	1: standing, walking	frontal	0.5–1	2 × 7.25 (bw 2.5)	C	a	(HR) = 86.9% accurate, absolute deviation (RR) = 2.3 bpm
[58]	RR	HR	5	1: laid/ seated	frontal, above	1	7.29 (bw 1.4)	C	a	deviation on lying subject depending on algorithm (HR) = 2.47–3.88 bpm and (RR) = 0.66–0.91 bpm
[59]	RR	HR	3	1/3: seated	frontal	2–2.5	2.4 (bw 0.5)	C	a	RMSE (RR) = 0.13 bpm and RMSE (HR) = 1.7 bpm
[60]	RR	HR	1 person + phantom	1: seated (phantom)	frontal	1, 2, 3, 4, 5	NVA 6100	phantom	a	deviation (RR) = 2.92% and (HR) = 4.24%
[81]	RR	HR	5	1-3: standing, walking	frontal	0.8–11 (through concrete)	NVA 6100	counting breath, “heart rate tester”	a	73–100 % accuracy in all experiments
[87]	RR		1	1: standing	frontal	3.3–9.3	0,4–4,4 (bw 4.36)	radar	a	n/a
[88]	RR		11 (2 babies)	1: laid, seated	frontal	1–4	7.29 (bw 1.5)	C	a	deviation (RR) = 0.96%, max. 0.170 bpm
[89]	RR		14 (2 babies)	1: seated	frontal	n/a	7.29 (bw 1.5)	C	a	max. deviation (RR) = 0.5 bpm
[90]	RR		model	1 model	2 radars sideways	1.5	2 × 4.7 (bw 3.2)	settings of model	a	97% accuracy
[91]	RR		14 (6 HI patients)	1: laid	diagonally above	1.25–2	8.7 (bw 1.5)	algorithm		detection of whether subjects were breathing: 98.64% accurate
[92]	RR		phantom	phantom	phantom	0.7	5.9–10.3	phantom data	a	94% accuracy breath classification
[93]	RR		15 (9 healthy + 6 persons with abnormal breathing patterns at night)	1: laid	above	0.5–2	7.29 (bw 1.5)	C	a	deviation (RR)= 6.12%
[111]		HR	5	1: seated	frontal	<1	6.8 (bw 2.3)	C	a	deviation (HR) = 1.05%
[112]		HR	22 (16 patients with atrial fibrillation)	1: laid	above	1–2	8.7	C		ICC healthy subjects (HR) = 0.856, r(HR) = 0.893
[113]		HR	3	1: seated, standing, back and forth movements	frontal, 2 in 45°	0.6	7.3 (bw 1.4)	C	a	RMSE (HR) between 1.05 bpm (seated without movement) and 15.21 bpm (standing, moving back and forth)
[114]		HR	8	1: laid	dorsal, sideways	through bed	4.1	C	a	compared to previous algorithm: valid evalation for 16–60% more cases possible
[115]		HR	2	2: walking	frontal	0.9–3.3	3 × 7.3 (bw 1.4)	C		(HR) = 85.93% accuracy
[119]		HR	7	1: standing	frontal	1–4.5	6–10	C	a	minimum error rate 4.6% in moving state and 2.25% in resting state
**Frequency-modulated continuous wave (FMCW) and UWB radar studies**										
[10]	RR	HR	1	1: seated	frontal, dorsal, sideways	0.5–2.5	60 (FMCW), 8.7 (IR-UWB; bw 1.5)	C		deviation in all experiments <7.4%
**FMCW radar studies**										
[61]	RR	HR	8	1: seated	frontal	1	120	C	a	accuracy (HR) = 90.54%, (RR) = 90.48% within ±2 bpm
[62]	RR	HR	10	1: laid	frontal, sideways, dorsal	2	24	C		deviation (HR) = 3.6% (86% correlation), (RR) = 9.1% (91% correlation)
[63]	RR	HR	20	1: seated	frontal	0.1–0.5	77 (UWB: bw 4)	C		deviation (RR) = 6.67%, (HR) = 2.9%
[64]	RR	HR	n/a	1: n/a	n/a	0.1–0.5	24	C	a	deviation (HR) = 0–6.3% and deviation (RR) = 0–9.5%
[65]	RR	HR	10	1/ 2: seated	frontal	1	77	C	a	deviation (RR) = 1.1 bpm and deviation (HR) = 6.8 bpm
[66]	RR	HR	3	1: seated	frontal	4.5	24 (bw 0.5)	C		90% in stop-breathing detection
[67]	RR	HR	n/a	n/a	n/a	n/a	35	n/a		n/a
[68]	RR	HR	11	2: seated	frontal	1–3	7.3	C		deviation within 1.5 bpm (RR)/3 bpm (HR)
[69]	RR	HR	5	1: seated	frontal, sideways, dorsal	1–3	5.8	C		error (RR) max. 0.8 bpm, error (HR) max. 3.1 bpm
[70]	RR	HR	11	1: standing, seated	frontal 90/60/120°	0.2–0.5	24	stethoscop, C	a	r(HR) = 0.93, (RR) n/a
[71]	RR	HR	3	3: seated	frontal	3.3–4.2	24/77	n/a		n/a
[72]	RR	HR	n/a	1/ 2: seated, standing	4 circling antennas	4	4 × 94	n/a		n/a
[73]	RR	HR	10 + simulation	1: seated	frontal, dorsal, left	1, 2	80	C + CO_2_		deviation (HR) = 8.09–18.94% depending on conditions, (RR) = 5.52–9.75%
[74]	RR	HR	10	1: seated	frontal	0.7–0.9	77	C	a	accuracy (HR) and (RR) = 93%
[75]	RR	HR	3	1: seated	frontal	1	77–81	C	a	r = 93.8%
[76]	RR	HR	n/a (dataset + probands)	1: walking, seated, standing	frontal, sideways, dorsal	0.3–1.5	77	C	a	deviation HR ± 4 bpm, best MSE (RR) = 1.04; 75% accuracy for arrythmia detection
[77]	RR	HR	5	1 (+interference person): laid	dorsal	0.2 (through bed)	60	C, CW-radar		>90% accuracy
[78]	RR	HR	n/a	1: seated	frontal	0.3	79	n/a	a	n/a
[79]	RR	HR	11	1: seated	frontal	0.8 - 1.5	77	C	a	deviation (RR) = 1.33% and deviation (HR) = 1.96%
[80]	RR	HR	6	1: seated	frontal	0.65	77	C	a	RMSE (HR) < 4 bpm, (RR) n/a
[116]		HR	16	1: standing	frontal	0.5	76–81	C	a	deviation depending on algorithm 1.86–10.06%
[117]		HR	5	1: seated + typing	frontal	0.28–0.7	77	C	a	deviation (HR) = 2.92%
[118]		HR	16	1: seated	frontal	n/a	60 and 77	C		smallest deviation (HR) = 0.02%, average (HR) = 6.4%
**Studies with unknown radar type**										
[13]	RR		16	1: laid	above, sideways	1	n/a	C (LG InnoteC)		deviation (RR) < 1 bpm

^1^ If not indicated otherwise: healthy adults. a = focused on algorithm; bpm = beats per minute; bw = bandwidth; C = contact measurement; CCC = concordance correlation coefficient; HR = heart rate; n/a = not available; ICC = intraclass correlation coefficient; ref. = reference measurement executed; radar (GHz) = used radar frequency—if not indicated otherwise, center frequency; RMSE = root mean squared error; RR = respiratory rate.

**Table 2:** Correlation between the frequency of the measured vital parameters and the frequency of the radar type used.

	CW Radar	UWB Radar	FMCW Radar	Radar Type Used n/s	Total
HR + RR	35	20	21	0	76
RR	6	8	0	1	14
HR	17	6	3	0	26
Total	58	34	24	1	

CW = continuous wave, UWB = ultra-wideband, FMCW = frequency-modulated continuous wave, n/s = not specified, HR = heart rate, RR = respiratory rate.

The number of test persons in the studies ranged from one test person [10,12,24,42,43,49,60,85,86,87,108] to 212 test persons [98,99]. Five of the experiments were conducted with children and babies [17,45,84,88,89], eleven with miscellaneous patient groups [5,17,21,39,41,51,88,89,91,93,112] and one with animals [38], while seven experiments additionally measured a model [37,42,44,60,85,97] and two exclusively measured a model [90,92]. In 74 studies [2,3,10,11,12,13,16,17,20,22,23,24,25,26,27,28,29,36,40,43,47,48,49,50,52,53,54,55,56,57,58,59,60,61,62,63,65,66,68,69,70,71,74,75,77,79,80,81,83,86,95,96,98,99,100,101,102,103,105,106,107,108,109,110,111,113,114,115,116,117,118,119,120], only healthy adults were examined, whereas 17 studies [14,15,18,19,30,31,32,34,35,37,46,64,67,72,76,78,104] did not contain any information on the test persons.

In 98 studies, one test person was measured per experiment [2,3,5,10,11,13,14,15,16,17,19,20,21,22,23,24,25,26,27,28,29,31,32,33,34,35,36,37,38,39,40,42,44,45,46,47,48,49,51,53,54,55,57,58,59,60,61,62,63,65,66,69,70,72,73,74,75,76,77,78,79,80,81,82,83,84,85,86,87,88,89,90,91,93,94,96,97,98,99,100,101,102,103,104,105,107,108,109,110,111,112,113,114,116,117,118,119,120]. In ten studies, experiments were conducted with two test persons simultaneously [16,40,48,52,56,65,68,72,81,115], and in seven studies, with three or more test persons simultaneously [16,48,50,56,59,71,81]. In four studies, persons employed to interfere with the signal (interfering persons) were additionally included [25,40,55,77]. Some studies comprised several experimental parts in which the number of test persons measured simultaneously varied [16,40,48,56,59,65,72,81]. In six studies, the number of test persons measured simultaneously was not clearly described [12,18,30,41,67,95].

The test persons’ positions relative to the radar were as follows: lying in 36 studies [2,3,5,13,17,23,28,29,31,32,33,34,44,45,50,51,53,55,58,62,77,82,84,88,91,93,94,99,104,105,106,107,110,112,114,120]; sitting in 60 studies [3,10,11,14,15,16,17,19,20,22,24,25,31,36,40,42,46,47,48,49,52,53,54,56,58,59,60,61,63,65,66,68,69,70,71,72,73,74,75,76,78,79,80,83,85,86,88,89,96,97,100,101,102,103,109,110,111,113,117,118]; standing in 14 studies [3,21,26,27,39,57,72,76,81,87,110,113,116,119]; standing with a pendular movement [113], sitting on an ergometer [14] and sitting and typing on a keyboard [117] in one study each; and running in six studies [58,77,82,109,110,115]. Where measurements were performed with static test persons after physical exercises, the positions assumed during the measurements were evaluated. In three studies, the test persons were behind a wall consisting of various types of stones [31,56,81] or earthquake debris [32]. In three further studies, the test persons were behind (acrylic) glass [29,84,104]. In five experiments, the test persons were measured through a mattress in a lying position [28,77,105,106,114], and in one study, they were measured through a chair in a sitting position [54]. In ten studies, it was not possible to obtain any information on the positioning of the test persons [12,18,30,35,41,43,64,67,95,108].

The radar used was positioned frontally to the test person in 81 studies [3,10,11,12,15,16,18,19,20,21,22,24,25,26,27,30,31,32,34,35,36,37,38,39,40,42,44,45,46,47,48,49,52,53,55,56,57,58,59,60,61,62,63,65,66,68,69,70,71,73,75,76,78,79,80,81,83,84,85,86,87,88,89,95,96,97,98,99,100,101,102,103,108,109,110,111,113,115,116,118,119], dorsally to the test person in 20 studies [10,12,17,22,23,28,31,42,47,52,54,62,69,73,76,77,84,86,95,114] and laterally to the test person in 21 studies [2,5,10,13,14,28,31,47,62,69,70,72,73,76,84,86,90,94,107,113,114]. In five studies, the measurements were performed from below [2,33,39,105,106], and in 15 studies, from above [3,5,13,29,50,51,58,82,84,91,93,99,104,112,120]. Multiple specifications of the positioning were possible due to the use of several radars, among other things. Four studies did not contain any information on the relative position of the radar [41,43,64,67].

The distance between the test persons and the radar varied between 0.15 and 30 m. In 72 studies, measurements were performed at a distance of up to 1 m [2,5,10,11,12,13,14,16,17,18,19,22,23,25,26,27,30,33,34,35,36,37,38,39,40,42,43,44,45,48,51,55,57,58,59,61,63,64,65,70,74,75,76,77,78,79,80,80,81,82,83,84,86,92,93,95,96,97,100,101,102,103,107,108,109,110,111,113,115,116,117,120]. In 39 studies, the distance was between 1 and 10 m [3,10,15,20,24,31,32,37,44,46,47,48,49,50,53,56,59,60,62,66,68,69,71,72,73,76,79,81,85,87,88,90,91,93,94,109,112,115,119], and in 3 studies, it exceeded 10 m [31,49,81]. A total of 13 studies did not contain any information on the distance [21,28,41,54,67,89,98,99,104,105,106,114,118].

The employed radars differed in the frequencies used. In five studies, radars with a central frequency in the range of <1 GHz were used [12,25,29,32,87]. In 57 studies, the radars employed used a central frequency in the range of 1–10 GHz [5,10,11,12,15,16,19,20,22,24,27,30,31,32,34,35,38,39,41,43,46,47,48,49,51,52,53,54,55,57,58,59,68,69,83,85,86,87,88,89,90,91,92,93,94,95,97,103,104,107,108,111,112,113,114,115,119]; in 36 studies, a central frequency in the range of 10–30 GHz was used [2,3,12,14,17,18,21,23,26,28,33,36,40,42,44,50,62,64,66,70,71,82,84,92,96,98,99,100,101,102,104,105,106,108,109,120]; and in 21 studies, frequencies > 30 GHz were used [10,37,61,63,65,67,71,72,73,74,75,76,77,78,79,80,108,110,116,117,118]. The frequency most frequently selected was 24 GHz. It was used in 29 studies [2,3,12,14,17,18,21,23,28,33,36,40,42,44,62,64,66,70,71,82,84,96,98,100,101,102,105,106,120]. In 16 studies, several radar frequencies or a frequency spectrum were used [2,5,10,12,32,33,39,51,53,71,75,87,92,104,105,108]. In two of these studies, different frequencies were used for measuring the heart and respiratory rates [2,39], whereas five studies did not contain any information on the central frequency used [13,45,56,60,81].

In 94 studies, the reference measurement was performed using contact electrodes [2,3,10,11,12,13,14,15,16,17,18,19,20,21,22,23,24,25,26,27,28,29,30,33,34,35,36,38,40,41,42,43,44,45,46,47,48,49,50,51,53,54,55,57,58,59,61,62,63,64,65,66,68,69,70,73,74,75,76,77,79,80,82,83,84,86,88,89,93,94,95,96,97,98,99,100,101,102,103,104,105,106,107,110,111,112,113,114,115,116,117,118,119,120]. This term included ECG electrodes and polysomnography systems, respiratory belts, pulse bands, finger sensors, pulse oximeters, etc. In six studies, manual recording of the applicable values was performed by the test person or another person [5,36,70,81,98,110]. In five of the studies, a measuring method already established in previous studies and based on a second radar or algorithm was used [39,52,56,87,91]. In two experiments, the test persons held their breath [18,32]. In three studies, a respiratory rhythm was set by a metronome for the duration of the experiment [15,85,103]. In one study, a CO_2_ measurement was used in addition to contact electrodes [73]. In studies where a phantom was employed, the phantom setting was used as a reference. Nine studies did not contain any information on the reference measurements [31,37,41,67,71,72,78,108,109]. In 64 sources, the focus was on the algorithm and signal processing [2,3,14,15,16,17,18,19,20,21,22,23,24,25,26,46,48,49,50,52,55,56,57,58,59,60,61,64,65,70,74,75,76,78,79,80,81,82,83,84,85,87,88,89,90,92,93,95,96,97,98,99,100,101,102,103,110,111,113,114,116,117,119,120]. This corresponds to 56% of the studies.

In 15 studies, the quantitative results of the experiments were reported by indicating the Pearson correlation coefficient (r-value) [2,11,17,23,28,30,35,39,42,52,71,76,94,106,112], in two studies by indicating an intraclass correlation coefficient (ICC) [5,112] and in two studies by indicating a concordance correlation coefficient (CCC) [45,53]. In seven studies, the quantitative evaluation was performed by indicating a root mean square error (RMSE) [17,28,59,80,82,84,113]. In 16 studies, the accuracy was indicated in percentage terms [14,19,30,43,47,57,61,74,77,81,86,90,105,107,108,115]. In 32 studies, the deviation was indicated as a percentage value [10,14,15,19,20,26,36,40,43,46,48,54,55,60,62,64,73,79,84,88,93,95,96,97,100,102,111,116,117,118,119,120]. The absolute deviation in beats per minute (bpm)/breaths per minute (bpm) was indicated in 24 studies [3,13,16,25,33,44,48,55,57,58,65,68,69,76,80,83,88,89,94,95,99,103,110,113]. For all the indicated data, the reference measurement was used as a reference point. Twelve studies either indicated semiquantitative data or did not focus on the quantitative acquisition of heart and respiratory rates [21,22,30,39,52,54,56,66,91,92,101,114], and 26 studies (partly) did not contain any information on the quantitative evaluation of the experiments [11,12,18,20,24,25,26,31,32,33,35,37,40,41,42,67,71,72,76,78,80,85,87,98,104,109]. 

A correlation coefficient r, according to Pearson, for the measurement of the respiratory rate by means of radar as compared to the reference measurement was indicated in ten studies [2,17,23,27,29,34,38,51,75,94]. 

The r-value was in the range of ≥0.99 in two studies [23,34], in the range of ≥0.95 in seven studies [2,23,27,34,38,51,94] and in the range of ≥0.9 for nine evaluations [2,23,27,29,34,38,51,75,94]. In two studies, the r-value was in the range of 0.8 to 0.9 [17,29]. There was no study with an r-value < 0.8. An r-value for measuring the heart rate was indicated in 14 studies [2,12,17,23,27,29,34,38,41,51,70,75,106,112]. There was no evaluation with an r-value ≥ 0.99. In five studies, the r-value was ≥0.95 [17,27,29,34,41], and in ten evaluations, it was in the range of ≥0.9 [17,23,27,29,34,38,41,51,70,75]. Two results showed an r-value in the range of 0.8 to 0.9 [12,112], and in two studies, there was an r-value < 0.8 [2,106]. The best correlation coefficients between the reference measurement and radar measurement were r = 0.99 for the respiratory rate [23,34] and r = 0.98 for the heart rate [34]. The lowest correlations were r = 0.83 for the respiratory rate [17] and r = 0.69 for the heart rate [2]. The underlying p-values of the studies were in the range of 0.01 to 0.05.

An intraclass correlation coefficient (ICC) was indicated in two studies [5,112]. In the given context, the ICC permits a quantitative assessment of the conformity of the measuring results of the radar and reference measurements [121,122].

In one study, an ICC was indicated for the respiratory rate [5]. In another study, an ICC was indicated for the accuracy of the heart rate measurement [112]. In both cases, the ICC was in the range of 0.8 to 0.9 [112]. In two studies, a concordance correlation coefficient (CCC) according to Lin was indicated [45,53]. In contrast to the ICC, which measures the relative conformity, the CCC assesses the absolute conformity [121,123,124]. With regard to the respiratory rate, the CCCs for both studies were in the range of 0.9 to 1 [45,53]. With regard to the heart rate results, the following values were determined: CCC = 0.9 to 1 and CCC < 0.8 [45,53].

In seven studies, the indication of a root mean square error (RMSE) was used for the evaluation [17,28,59,80,82,84,113]. In two studies, the temporal deviation [125] of the measured interval from the reference interval of respiration or the R waves of the heartbeat was used for this purpose [28,82]; in five studies, the absolute deviation in bpm [17,59,80,82,113]; and in one study, the deviation indicated in percentage terms [84]. With regard to the respiratory rate (RR), the measured temporal deviations resulted in the following values: RMSE (RR) = 26.07 ms [82] and RMSE (RR) = 200 ms [28]. With regard to the heartbeat, the following value was determined: RMSE = 24 ms [28]. The absolute deviations of the RMSE in bpm are included in the section on the absolute deviation, whereas the percentage deviations calculated by means of the RMSE are included in the section on the relative deviation.

Quantitative data on the percentage deviations of the respiratory rate, or, reciprocally, data on the accuracy (percentage data as a measure of the accuracy of the respective results as compared to the results obtained using reference measurements) were available for 27 of the studies [10,14,15,19,30,36,40,43,46,47,48,55,60,61,62,63,64,73,74,77,79,81,84,86,88,90,93]. Since this review focuses on general applicability, the maximum deviations of the study results were considered when specifying a value range. Recent studies have indicated that a 5% deviation in heart and respiratory rate measurements may be acceptable in remote monitoring scenarios [126]. Since our review reports the deviation rates observed in various studies and highlights the need for further research to improve accuracy and address confounding variables in radar-based vital parameter measurement, we chose the given turnpoint of the literature and suitable neighboring values. In three studies, the maximum deviation of the respiratory rate was <1% [30,55,88]. This approximately corresponds to every tenth study indicated (11%). In ten experiments, the deviation was <5% [14,15,19,30,43,55,60,79,88,90]. This approximately corresponds to every third study (37%). In 23 experiments, the measured deviation was <10% [10,14,15,19,30,40,43,46,48,55,60,61,62,63,64,73,74,77,79,84,88,90,93]. This corresponds to 85% of the studies. In four studies, the maximum deviation was >10% [36,47,81,86]. This approximately corresponds to every sixth study (15%). Two studies showed partial results with a maximum deviation >20% [47,81]. This corresponds to 7% of the studies.

Quantitative evaluations of the percentage deviation of the heart rate/the accuracy were available for 39 studies [10,14,15,19,20,26,30,36,43,46,47,48,54,57,60,61,62,63,64,73,74,77,79,81,95,96,97,100,102,105,107,108,111,115,116,117,118,119,120]. In three of the measurements, the maximum deviation of the heart rate was ≤1% [14,108,118]. This corresponds to less than every tenth study (8%). In 19 experiments, the maximum deviation indicated was <5% [14,15,19,20,30,43,46,48,60,62,63,79,96,97,107,108,111,117,119]. This corresponds to half of all studies (48%). In 34 experiments, the maximum deviation indicated was <10% [10,14,15,19,20,26,30,36,43,46,47,48,54,60,61,62,63,64,73,74,77,79,95,96,97,102,105,107,108,111,117,118,119,120]. This corresponds to 87% of the studies. In five studies, the maximum deviation was >10% [47,57,100,115,116]. This corresponds to 13% of the studies. Two studies showed partial results with a deviation > 20% [54,81]. This corresponds to 5% of the studies. With regard to the heart rate measurement, the percentage deviation ranged from 0.02% [118] to 39.4% [54], and with regard to the respiratory rate measurement, from 2% [30] to 27% [81].

**Figure 3:**
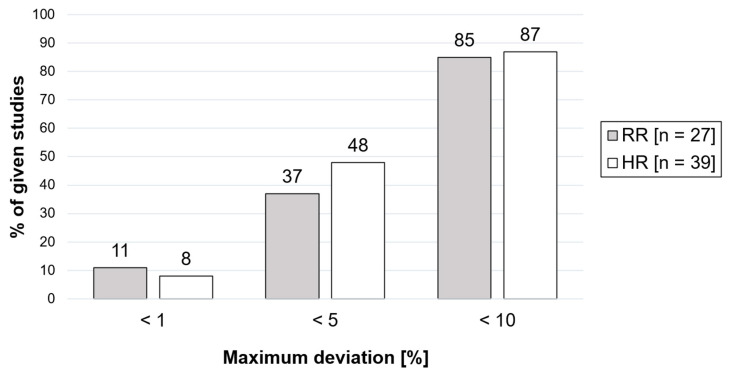
Percentage of studies within a maximum deviation of 1/5/10% for the heart rate (HR) and the respiratory rate (RR).

Figure 3 shows the percentage of studies within a defined maximum deviation range (indicated as a percentage value) of the heart rate (HR) and the respiratory rate (RR).

**Figure 4:**
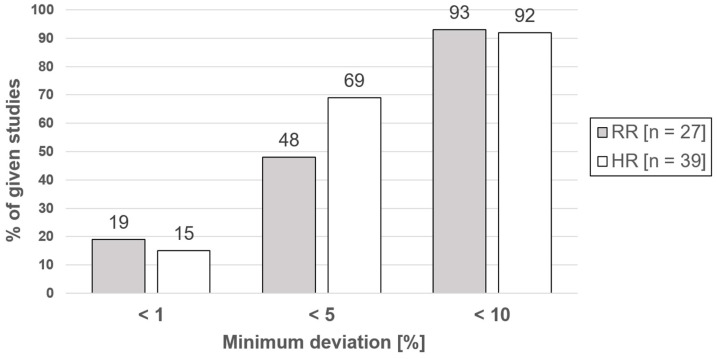
Percentage of studies within a deviation of 1/5/10% at maximum for the heart rate (HR) and the respiratory rate (RR), taking into an account the results deviating least from the control values.

Figure 4 shows the percentage of studies where the best measured result was within a defined maximum deviation range (indicated as a percentage value) of the heart rate (HR) or the respiratory rate (RR).

The absolute deviation in breaths per minute (bpm) was indicated in 20 studies [3,13,16,17,44,48,50,57,58,59,61,65,68,69,76,82,83,88,89,94] and was <1 bpm in 11 studies [13,44,48,50,58,59,69,82,88,89,94]. This corresponds to approximately half of the studies. In all studies, the deviation was <5 breaths per minute (bpm) [3,13,16,17,44,48,50,57,58,59,61,65,68,69,76,82,83,88,89,94]. The measured deviation ranged from 0.13 bpm [59] to 2.3 bpm [57].

The absolute deviation in beats per minute (bpm) was indicated in 20 studies [16,17,25,44,48,50,55,58,59,65,68,69,76,80,83,95,99,103,110,113] and was <1 bpm in two studies [13,25]. This corresponds to every tenth study. In 16 studies, the deviation was <5 bpm [16,17,25,44,48,50,55,58,59,68,69,76,80,83,103,110], and in 19 studies, it was <10 bpm [16,17,25,44,48,50,55,58,59,68,69,76,80,83,95,99,103,110,113]. The measured deviation ranged from 0.8 bpm [83] to 15.21 bpm [113].

**Figure 5:**
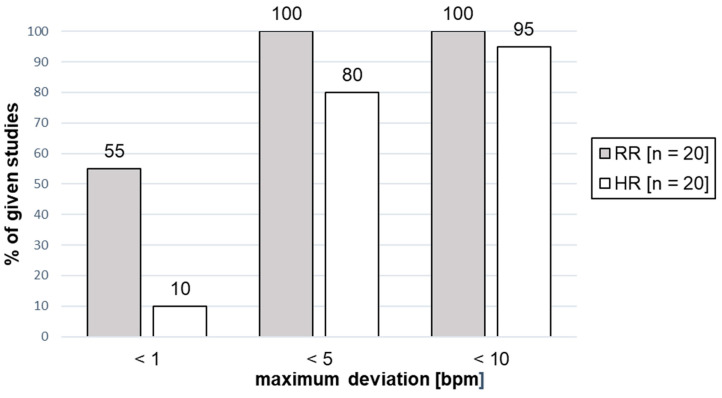
Percentage of studies within a maximum deviation of 1/5/10 beats/breaths per minute (bpm) for the heart rate (HR) and the respiratory rate (RR).

Figure 5 shows the percentage of studies within a defined maximum deviation range (indicated in bpm) of the heart rate (HR) or the respiratory rate (RR).

In 39 studies, the data on the heart and respiratory rates were quantitatively comparable [2,14,15,16,17,19,23,27,28,29,30,34,36,38,43,44,45,46,47,48,50,51,52,53,58,59,60,61,62,63,64,65,68,69,73,74,77,79,83]. In *n* = 23 of these studies, the respiratory rate measurement was more accurate than the heart rate measurement [2,17,23,30,34,38,43,44,50,51,52,53,58,59,60,64,65,68,69,73,74,77,79]. This corresponds to 59% of the studies.

## 4. Discussion

In order to adequately answer the questions in connection with this systematic literature review, the current fields of application, advantages, disadvantages and problems described in the current literature and possible future prospects of radar-based vital parameter measurement were placed in the context of the preceding quantitative evaluation and then discussed in this context. The described fields of application of non-contact measurement of heart and respiratory rates can be assigned to three categories: “monitoring”, “screening and diagnostics” and “emergency medicine”.

Monitoring of a person may be necessary in an acute situation or in the long term. Acute fields of application examined in the reviewed literature include monitoring of the cardiac rhythm [5] and intracorporal movement monitoring of the bladder, vessels and the heart [46]. Monitoring of infectious patients in ambulances [2] and monitoring of neonates and babies [34], e.g., for the purpose of preventing sudden infant death syndrome (SIDS) [97], have also been described. Kebe et al. depicted the possibility of short-term monitoring of competitive athletes with regard to physical and psychological stress responses [1]. In the field of non-contact long-term monitoring, general home monitoring of elderly people [50] and monitoring in case of suicide risks in the geriatric population and in prison inmates [66] have been examined. Khan et al. could not identify any risks to health, so daily use was declared possible [127].

In the category of “screening and diagnostics” by means of radar technology, a distinction could be made between acute and chronic problems. Rong et al. referred to the possibility of screening for acute infectious diseases in order to reduce the risk for health care workers [113]. In the reviewed literature, screening at airports as a measure to prevent the spread of infectious diseases has been examined as well [27]. The measurements conducted by Kim et al. focused on the prevention of accidents by diagnosing arising fatigue [107]. Several authors [1,128,129,130] examined the obstructive sleep apnea syndrome (OSAS) as a chronic indication, including the determination of sleep stages by means of radar-based vital parameter measurement [131].

In the category of “emergency medicine”, Chen et al. examined the efficacy in search and rescue operations to detect buried earthquake victims through rubble and debris [32].

In these fields, radar technology is considered the most effective method of non-contact vital parameter measurement [12]. Due to the advantages of detection/acquisition over a physical distance, it can be used both for supporting already existing technologies [104] and as an independent measuring method [132]. Thus, radar technology functions independently of environmental factors such as light, temperature and noise level, and does not affect daily routines through contact electrodes [97]. The measurement is performed by detecting movements of the body surface [104], even through clothing [31]. During the measurement, the privacy of the person to be measured thus remains unaffected [105], and the risks of contact measurement are avoided. This factor is particularly important when conventional measurements are painful, unpleasant or unsafe [45,50], and, as became obvious during the COVID-19 pandemic, it has also gained importance with regard to preventing the transmission of infections.

Disadvantages of the measurement by means of radar technology were not described in the reviewed literature [127]. Possible concerns that radiation from the radar equipment may harm the test person are unjustified, since this equipment has a higher degree of radiation safety than commercial mobile phones [1].

The greatest difficulties of measurement by means of radar technology are inaccuracies caused by background noise [32] (particularly vibrations) and so-called random body movements (RBMs) [101]. These difficulties require mechanisms in order to avoid them [1].

Two components are of vital importance for the general use of radars: hardware and digital signal processing. The attempt to counter RBMs and signal problems using hardware alone has proven to be impractical [15]. As early as 2002, the trend towards digital signal processing emerged [133]. Thus, more than half of the studies considered focused on algorithms and software (56%). From a medical point of view, however, the software component of radar can be problematic. For example, algorithms used for improving the signals of heart rate measurements are based on the assumption that the heart rate does not change suddenly [52], or they suspend the measurement in the superimposed interval altogether [52,105]. In general, but as was specifically mentioned in Khan et al [52] and Higashi [105], the algorithms for heart rate measurement assumed stable heart rates over short intervals, leading to challenges in situations where the heart rate changes rapidly, such as during intense physical activity or acute medical events. Some algorithms may also suspend measurements during periods of high signal interference or noise, potentially missing critical data. The impact of these assumptions and methods could include reduced accuracy or reliability of heart rate measurements in dynamic or complex scenarios.

With regard to the hardware component, the type of radar equipment is a decisive factor. In addition to performance, the size and easy handling of the equipment are particularly important for its usability [49]. Due to its simplicity and compact size, CW radars are used most frequently, especially in older studies (refer to Table 2). FMCW radars are mainly found in more recent studies (refer to Table 1). Among other things, they are used to discriminate different signals [21]. As multiple-input multiple-output (MIMO) radars, they are suitable for simultaneous monitoring of several test persons [116]. Continuous wave (CW) radar systems are known for their simplicity and cost-efficiency, but they struggle with complex signal processing in dynamic environments. In contrast, frequency-modulated continuous wave (FMCW) radar systems, featured in recent research, offer better accuracy and signal discrimination, making them ideal for high-precision and multi-person monitoring. However, their drawbacks include technical complexity and potential higher costs, limiting their use in simpler applications.

In addition to the difficulties posed by RBMs and background noise as far as the metrological side is concerned, the patient’s abdominal circumference negatively correlates with the precision of the measurement as well [130]. With regard to the impact of the positioning of the test person relative to the radar, there are partly contradictory statements as to the measurement from a lateral position [55,76]. There is agreement on the fact that the best precision is achieved with frontal or dorsal positioning of the patient in a lying position and at a short distance [53,55,76,99]. This is also reflected in quantitative evaluations. In most of the studies, a single sitting or lying test person was measured from the frontal or dorsal side in an interference-free area. Here, more than half of the studies examined distances < 1 m. These test conditions minimize the confounding variables of RBMs and interference, thus leading to more precise results.

Looking at the percentage deviation of respiratory rate and heart rate in Figure 2, >85% of the studies achieved deviations < 10%. In 48% of cases, a deviation of <5% could be measured for the heart rate, and in 37% for the respiratory rate. A deviation of <1% was achieved by 11% of the studies that measured the respiratory rate and 8% of the studies that measured the heart rate. Looking at the best results achieved in the study in Figure 4, >90 % of the studies achieved deviations < 10 %. In addition, 69% of the studies that measured heart rate achieved results with deviations < 5%. When measuring the respiratory rate, this result was achieved in 48% of the studies. A deviation of less than 1% was achieved in 19% of the studies measuring respiratory rate and in 15% of the studies measuring heart rate. A look at the absolute deviations in Figure 5 shows that 100% of the studies achieved a deviation of <5 breaths per minute, and in 55%, this deviated by less than 1 breath. For heart rate, 95% remained below a deviation of 10 beats/min and 80% below a deviation of 5 beats/minute. Finally, 10% of the studies achieved an absolute deviation of <1 heartbeat/minute.

In the literature reviewed, the heart rate is generally regarded as the vital parameter that is more difficult to measure [5]. This is due to the extent of movements connected with the respiratory rate, which is therefore easier to detect. The overlapping of the heartbeat with respiration also plays a part in this context. When looking at the results, however, it is noticeable that the heart rate could be measured more precisely than the respiratory rate in 41% of the studies with comparable values for both parameters. In Figure 2 and Figure 3, the expected discrepancy in measurement precision between the heart and respiratory rates even changed to the opposite. In these cases, the percentage of studies below a maximum deviation for the respiratory rate is lower than that for the heart rate for most of the values (cf. Figure 2 and Figure 3). In Figure 4, however, the determination of the respiratory rate shows a significantly better precision. This phenomenon can be explained by two aspects. Firstly, the heart rate in a healthy test person is significantly higher than the respiratory rate. As a result, a deviation by one breath has a much more noticeable impact than a deviation by one heartbeat when it comes to indicating the deviation as a percentage value. Secondly, the trend towards focusing on the algorithm becomes apparent here: Some algorithms are primarily intended to determine the heart rate. Here, the respiratory rate is determined as a secondary value. The expectation that the heart rate would be more difficult to determine resulted in a change to the opposite so that—contrary to the expectations of the current literature—the heart rate measurement yielded better results than the respiratory rate measurement in 41% of the studies.

Most of the studies show good to very good measuring results. The described field of application for radar-based vital parameter measurement is also wide, provided there is no contraindication. Nevertheless, this type of measurement has only been used to a small extent in (clinical) everyday practice. RBMs and interferences are still the central problem of measurement optimization. It is certainly a decisive factor as well that the ECG is already a long-established, well-functioning, easily applicable technology covering many fields of application of radar-based vital parameter measurement. This makes it more difficult for new technology to become accepted.

Despite the large number of studies included, this review has its strengths and weaknesses. A weakness of any review is a possible lack of studies and information due to the selection of the databases to be included and lacking data in the studies, or as a result of language barriers. This was countered, among other things, by the inclusion of several databases and application of the PRISMA criteria [9]. However, the large number of studies included indicates that this systematic review reflects the current scientific state of knowledge. Another factor that needs to be considered is publication bias. This leads to an elimination of the approaches that do not work and could, thus, insinuate that the technology is easily applicable. Studies showing the non-applicability of this technology will naturally be published less frequently. The limited comparability of the publications due to their heterogeneous study designs and the differing selection of parameters used to indicate the quality of the measurement also make it difficult to make general statements. This problem is aggravated by the fact that many studies examined only a few people, compromising the statistical significance and generalizability of the results. Furthermore, the optimized testing conditions frequently chosen to reduce interference, like the quiet setting of the probands, may not necessarily reflect real-world operational conditions, limiting the transferability of these findings. Therefore, the conclusions drawn from these studies should be considered in light of their specific nature and potential limitations in their experimental design, sample size and statistical analysis.

Despite the difficulties and problems mentioned above, the field of radar-based vital parameter measurement has a clear legitimation and a promising perspective. The ever-growing interest is reflected in a rapidly increasing number of publications over the years, as displayed in Figure 2. Its many advantages and fields of application lie, in particular, in areas where conventional vital parameter measurement is stretched to its limits. The field to be mentioned first and foremost in this respect is the field of non-contact applications. These applications comprise everyday situations where it is important to maintain privacy, e.g., in the case of permanent (home) monitoring. However, there are also situations in emergency and disaster medicine where contact is not safe for medical personnel or test persons (e.g., in case of infections, burns) or access to patients is difficult (e.g., in case of natural disasters), which also constitute possible fields of application. By linking radar-based vital parameter measurement to other future-oriented technologies such as drones, it will be possible to further extend the field of application in disaster and emergency medicine. To this end, it will be crucial to solve the problem of interferences and RBMs by further optimizing the algorithms and the hardware and to concentrate research on areas requiring non-contact measurement.
